# Self-Reported Socio-Economic and Psychological Burdens for Caregivers of Patients Undergoing Dialysis: A Cross-Sectional Study

**DOI:** 10.7759/cureus.80353

**Published:** 2025-03-10

**Authors:** Mandreker Bahall, Anuka D Harry, Anjali Kisseur, Vidal Ramdass, Dominique Gransaul, Sydney Alexander, Sacha Nandlal, George Legall

**Affiliations:** 1 Caribbean Centre for Health Systems Research and Development, The University of the West Indies - St. Augustine Campus, St. Augustine, TTO; 2 School of Medicine, Faculty of Medical Sciences, The University of the West Indies - St. Augustine Campus, St. Augustine, TTO; 3 Faculty of Medical Sciences, The University of the West Indies - St. Augustine Campus, St. Augustine, TTO

**Keywords:** burden, challenges, dialysis, primary caregiver, quality of life

## Abstract

Background

Patients undergoing dialysis require intensive treatment and supportive care, which affects their caregivers physically, socially, economically, and psychologically. However, this topic has been largely underexplored.

Objectives

This study aimed to examine the socio-economic and psychological burdens experienced by primary caregivers of patients undergoing dialysis.

Method

This cross-sectional study was conducted over 12 weeks using purposive sampling of primary caregivers of patients undergoing dialysis at two public and two private dialysis centers in Trinidad and Tobago. The questionnaire collected data on socio-demographics, economics, and selected psychological issues. Selected global health status questions adapted from the World Health Organization Quality of Life Brief Version were included. Participants were assured of confidentiality and anonymity. Verbal consent was obtained by completing an online questionnaire. The collected data were entered into a Statistical Package for the Social Sciences spreadsheet. Data analysis included hypothesis testing at a 5% level of significance using chi-square tests of association and analysis of variance.

Results

The final sample comprised 150 participants (response rate: 100%). The majority of caregivers were aged 46-55 (n = 44, 29.3%), women (n =82, 54.7%), and had attained tertiary-level education (n = 82, 54.7%). Most caregivers were the patient's son or daughter (n = 53, 35.3%), lived with the patient (n = 83, 55.3%), and were employed full time (n = 104, 69.3%). More than half (n = 79, 52.7%) reported having at least one health condition, with 40% (n = 61) reporting "getting sick easily" after becoming a caregiver. The majority experienced psychological symptoms of feeling nervous, anxious, or on edge (n = 115, 76.7%). Caregivers reported feeling depressed (n = 49, 32.7%), experiencing burnout (n = 101, 67.8%), and having suicidal thoughts (n = 10, 6.7%). Caregivers spent between one (n = 4, 2.7%) and four (n = 30, 20.0%) days a week taking patients for dialysis treatments, with the majority (n = 80, 53.3%) spending three days weekly. Travel time to dialysis centers ranged from 45 minutes (n = 46, 30.7%) to three hours (n = 5, 3.0%). Most caregivers also reported difficulty having a good work-life balance (n = 106, 70.7%), inability to attend social events (n = 93, 62.0%), reduced personal time (n = 86, 57.3%), and the need to adjust their work hours (n = 99, 66.0%). The majority also experienced economic hardships such as worrying about finances (n = 102, 68.0%). Caregivers also wished other family members contributed financially to patient care (n = 107, 71.3%), especially because the majority (n = 99, 66.0%) were unable to afford to send the patient to a nursing home. Nearly half (n = 66, 44%) of caregivers rated their quality of life (QoL) as "less than good"and more than half (n = 99, 66%) felt that their life lacked meaning.

Conclusion

Caregivers spend a considerable amount of time with patients, which affects them biologically, socially, economically, and psychologically. More than half of caregivers had challenges in maintaining a work-life balance. The majority were worried about finances (n = 102, 68.0%). Participants experienced anxiety (n = 116, 76.7%), burnout (n = 102, 68.0%), depression (n = 49, 32.7%), and suicidal thoughts (n = 10, 6.7%). These findings underscore the need for targeted intervention to support caregivers and improve their overall QoL.

## Introduction

End-stage renal disease (ESRD) is a common condition, accounting for a global prevalence of 4.902-7.083 million [1]. In Trinidad and Tobago, the prevalence is 875 per million population [2], one of the highest globally, and is reported in the annual report from the United States Renal Data System [3]. Dialysis is the primary treatment for preventing or minimizing ESRD-related complications. Caregivers of patients undergoing dialysis reported experiencing poorer quality of life (QoL) [4], psychological consequences [5], anxiety (27.8%), and depression (11.4%) [6]. According to Sajadi et al. [5], caregivers experience psychological pressure, isolation, and neglect, which adversely affect their mental and physical health. Ibrahim et al. [7] identified common caregiving burdens, including physical, psychological, social, and financial challenges. Factors such as caregiver demographics, disease-related variables, interpersonal relationships, social support, and psychological factors (e.g., depression and anxiety) contribute to this burden [8].

No studies have examined the socio-economic and psychological impacts on caregivers of patients undergoing dialysis in the Caribbean. This study aims to explore the socio-economic and psychological impacts on caregivers of patients undergoing dialysis, identify associations, and determine predictors. Given the global increase in ESRD cases [9], initiatives that support caregivers are increasingly crucial for understanding and improving their well-being. 

## Materials and methods

This cross-sectional study was conducted over 12 weeks (January to March 2024), due to time constraints and limited resources, and utilized purposive sampling to recruit the primary caregivers of patients undergoing dialysis. Trinidad and Tobago, a small resource-limited country, with 1.5 million people [10], has 14 dialysis centers, across its four health authorities. However, data were collected from two public and two private dialysis centers. The study sample was calculated as 150 participants using the formula below:

Sample Size = (Z value)^2^ *((p (1-p)) / (Margin of Error)^2^)

Purposive sampling was employed to ensure diverse caregiving experiences and viewpoints. Inclusion criteria involved caregivers aged 18 years or older and those serving as the primary caregivers for more than 50% of the time. Family members or individuals not directly involved in the patient's care were excluded. In healthcare, a primary caregiver is defined as any individual responsible for identifying, preventing, or treating an illness or disability [11]. Potential participants were assured of the anonymity and confidentiality of all information, briefed on the aim and nature of the study, and informed of their right to withdraw at any time. Participants who provided written consent were eligible to participate after fulfilling the inclusion and exclusion criteria. A self-made questionnaire was used to collect data on socio-demographics (27), economic status (six), social status (seven), and selected psychological issues (15). Questionnaires were distributed to caregivers present at the dialysis centers and entrusted to patients to be forwarded to their primary caregivers, or through an online platform.

Completed questionnaires (see Appendix) were stored in secure lockers, whereas digital data collected via Google Docs were accessible only through password-protected devices. Only the study group members and supervisor had access to view, edit, or comment.

The questionnaire included patient socio-demographics, dialysis treatment and duration, and caregiver characteristics, including age, sex, employment status, ethnicity, education level, and health conditions. These factors were examined in relation to economic, social, and psychological consequences. Descriptive statistics included frequency and percentage distribution tables, means, and standard deviations. Inferential analysis was conducted using 95% confidence intervals and hypothesis testing (chi-square tests of association and analysis of variance). All hypotheses were tested at a 5% level of significance.

Global health status scores were identified using four selected questions: “How would you rate your quality of life?”, “Are you satisfied with your health?”, “How much do you enjoy life?”, and “To what extent do you feel your life is meaningful?” Information is presented in tables and bar graphs, and percentages are categorically determined based on the social, economic, and psychological impacts on caregivers.

Ethical approval was obtained from the Ethics Committee of the University of the West Indies, Trinidad and Tobago, on December 13, 2023 (Ref: CREC-SA.2416/11/2023), and the Eastern Regional Health Authority and North Central Health Authority of Trinidad and Tobago on February 15, 2024, and March 15, 2024, respectively. All the participants voluntarily provided written informed consent to participate in this study. Participants were notified of the confidentiality of the data and their right to withdraw from the study.

## Results

Socio-demographics

**Figure 1:**
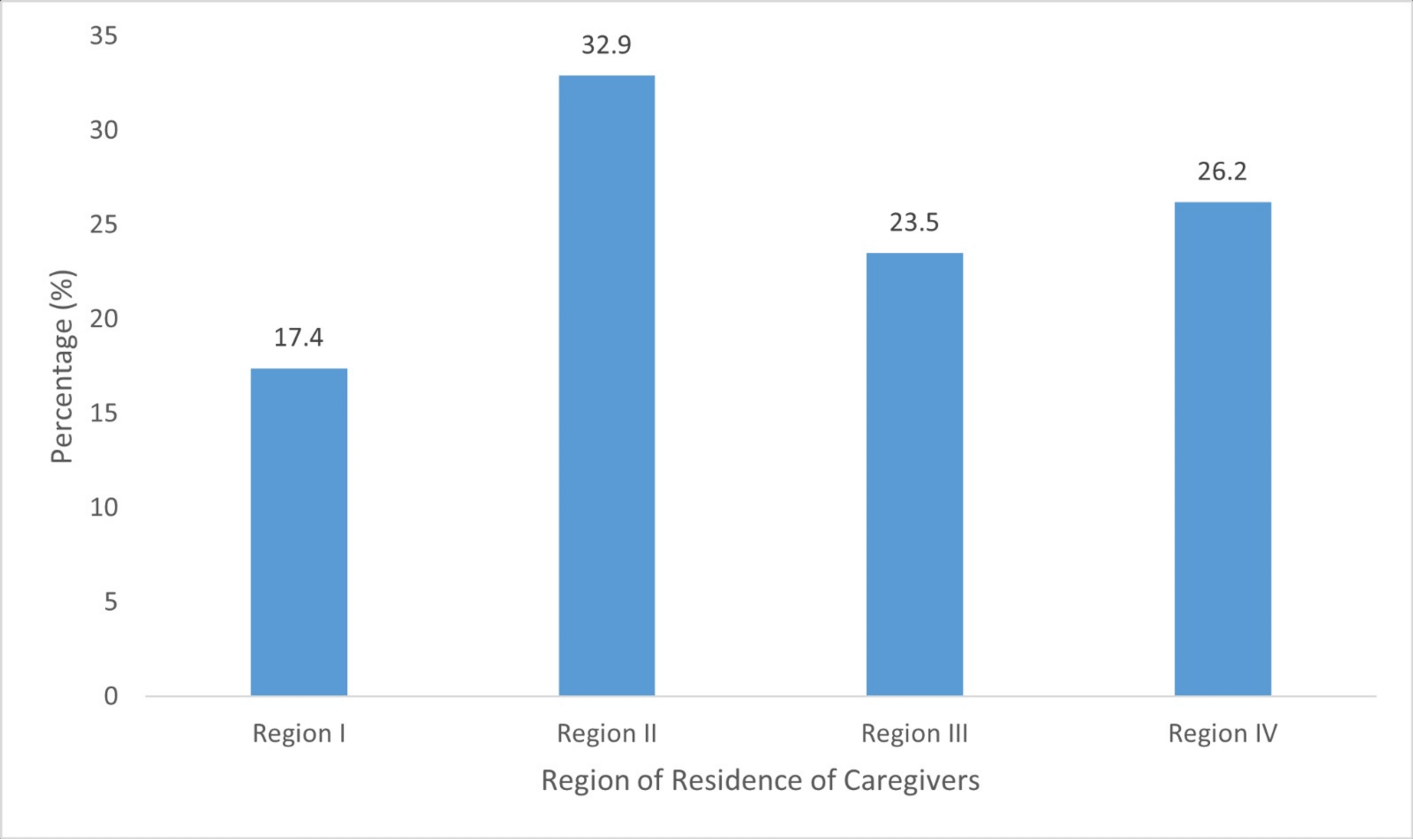
Health region of residences of caregivers

Data were collected from 150 caregivers from two public and two private dialysis centers, in four health regions. Figure 1 presents the respective percentage distributions.

Patient and caregiver characteristics

**Figure 2:**
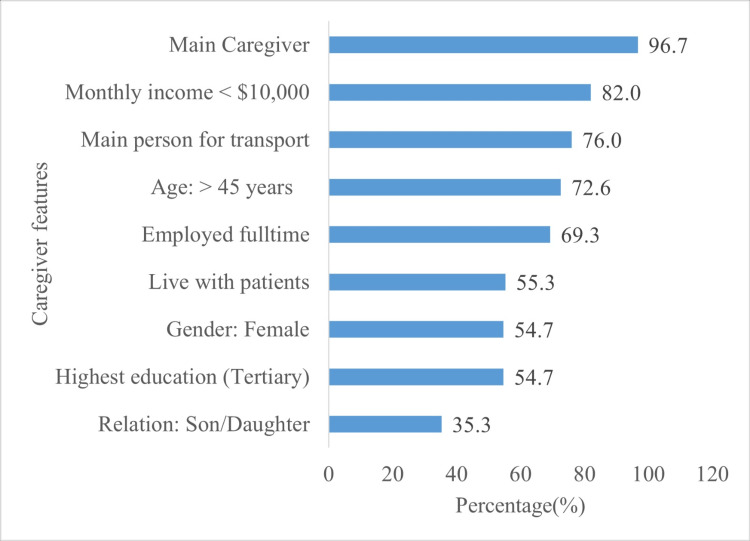
Primary caregiver characteristics

The majority of patients were men (n = 82, 54.7%) and belonged to the 46-75 age group (n = 113, 75.3%). Most caregivers were either the sole or primary caregivers. Other caregiver characteristics included being female, over 45 years of age, employed full time, and having at least a tertiary-level education. Moreover, more than half of the caregivers lived with the patient (Figure 2). Caregiver-patient relationships were children of the patients (n = 53, 35.3%), spouses (n = 39, 26.0%), and siblings (n = 20, 13.3%; Figure 2).

Age comparisons between patients and caregivers

**Figure 3:**
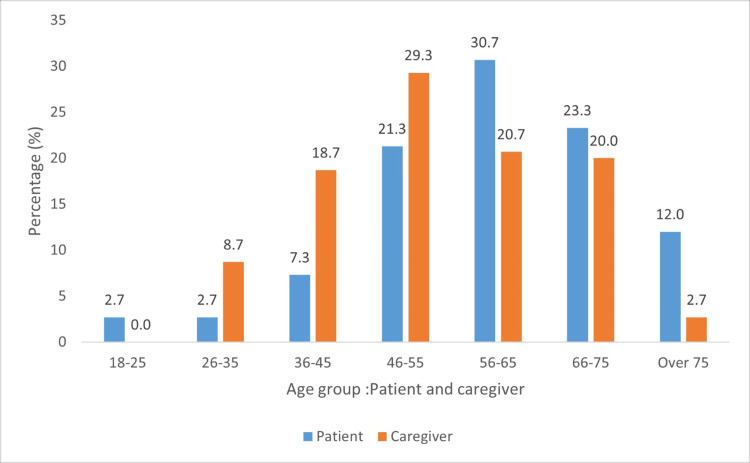
Age groupings of patients and caregivers

Age differences were mainly identified in the 26-35 and 56-65 age groups. The majority of caregivers were in the productive age range of 26-65 years (Figure 3).

Health conditions of caregivers and patients

**Figure 4:**
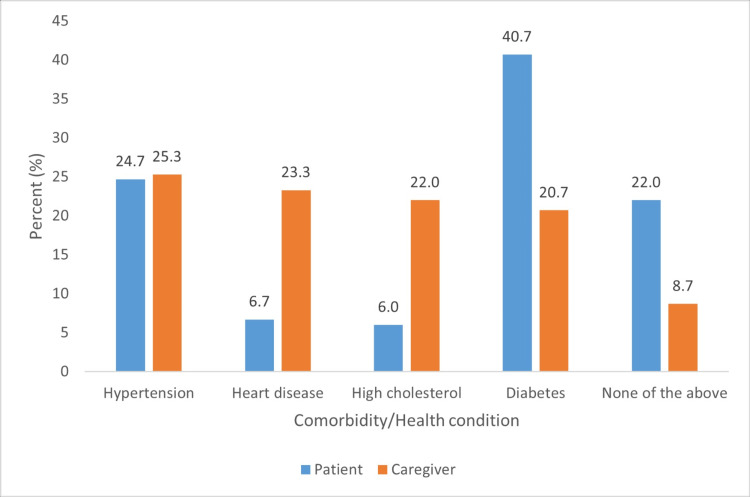
Selected health conditions (caregivers) and comorbidities (patients undergoing dialysis)

Eighty (52.7%) caregivers reported having at least one health condition, and six (7.5%) reported having four (Figure 4).

**Figure 5:**
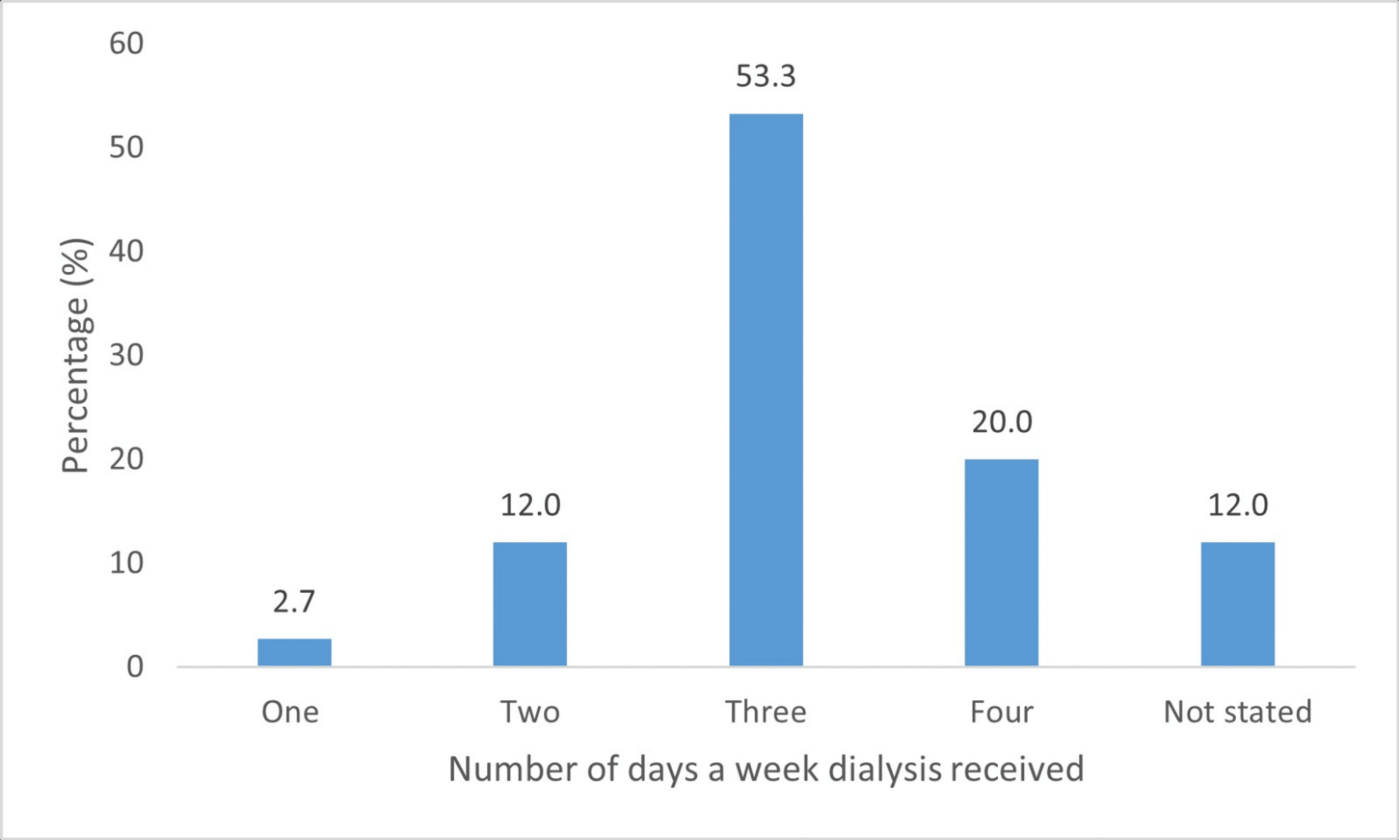
Days to dialysis centers weekly

Patients were taken to receive dialysis between one (n = 4, 2.7%) and four (n = 30, 20.0%) times weekly (Figure 5).

Dialysis sessions, travel time, and time with patient/exhaustion and sickness

Reported travel times from patients’ homes to dialysis centers ranged from as little as 45 minutes (n = 30, 20.0%) to three hours (n = 3, 2.0%). Some caregivers answered that they "drop the patients off and leave" (n = 58, 38.9%), others remain "sometimes" (n = 55, 36.7%), and about one-fifth of caregivers remain with the patient "always" (n = 29, 19.5%).

Caregivers reported "getting sick easily" (n = 61, 40.6%) after they became a caregiver and experiencing burnout or exhaustion from stress (n = 101, 67.3%). Some claimed to have had cause to visit a doctor for a health check since being caregivers (n = 32, 21.3%), whereas others reported an increase in alcohol consumption (n = 36, 24.0%).

Social consequences of caregivers

Table 1 presents the seven items used to measure self-reported consequences with the percentage of caregivers who answered "Yes" to each item.

**Table 1:** Frequency and percentage distribution of social consequences among caregivers

Variable	Rank	n	%
Hard to have a good work-life balance	1	106	70.7
Unable to attend many social events taking care of patient	2	93	62.0
Experienced judgment from others due to caregiver's role	3	66	44.0
Taking care of patients affected the closest relationships	4	57	38.0
I have good social support from family and/or friends	5	54	36.0
Spent less time on hobbies due to caregiving	6	35	23.3
I feel lonely as a result of taking care of the patient	7	24	16.0

Lower scores indicate less adverse social consequences.

Cronbach alpha = 0.664: Interpretation: Questionable reliability

The most-reported social consequence of caregiving was "Finding it difficult to maintain a good work-life balance" (n = 106, 70.7%), which was significantly associated with the caregiver’s relationship to the patient (chi-square = 11.336; df = 6; p = 0.045). The second most common consequence was "Unable to attend many social events while taking care of patients" (n = 93, 62.0%), which was significantly associated with the caregiver's age group (chi-square = 8.333; df = 3; p = 0.039).

Economic consequences of caregivers

The most reported economic consequence of caregiving was "I wish other members of the family would help contribute financially to taking care of the patient" (n = 107, 71.3%). The least reported was "I feel that I cannot sufficiently afford to pay for food, clothes, or vacations for myself or my family, owing to caregiving for the patient" (n = 83, 55.3%). Others included "wishing other family members would contribute financially"(n = 107, 71.3%), "having to worry about finances"(n = 102, 68.0%), and* *"having had to change job or work hours" (n = 99, 66.0%).

Psychological consequences for caregivers

**Figure 6:**
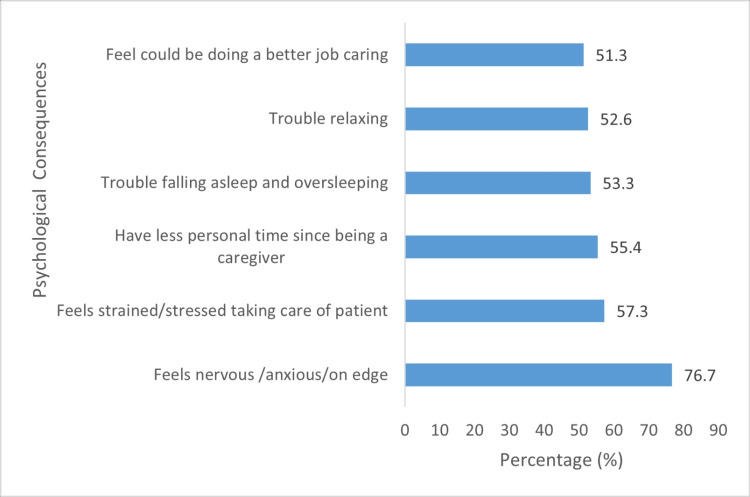
Prevalence of psychological consequences of caregiving

The possible scores for the psychological consequences of providing care ranged from zero to four. Total scores (for all 15 items) ranged from 0 (n = 7, 4.7%) to 60 (n = 3, 2.0%). The mean was 32.8 (SD = 14.36), mode 38 (n = 9), and median 36. The majority experienced "feeling nervous, anxious or on edge" (n = 115, 76.7%). Figure 6 details the six items (out of 15) with the highest percentages of caregivers answering "Often" or "Everyday." Some caregivers reported feeling "down, depressed or hopeless" (n = 49, 32.7%), while others reported having "self-harm or suicidal thoughts" (n = 10, 6.7%).

Caregiver's age (p = 0.006) and monthly income (p = 0.028) were the only variables significantly associated with mean stress scores (Figure 6).

Health status indicators

Almost half of caregivers (n = 66, 44.0%) rated their quality of life to be "less than good." Some caregivers responded either "Rarely" or "Not at all" when asked, "To what extent do you feel life is meaningful"(n = 99, 66%; Table 2).

**Table 2:** Questions related to health status QoL: Quality of life

QoL questions	Leading response	n	%
How would you rate your quality of life?	Less than good	66	43.0
Are you satisfied with your health?	No	84	56.0
How much do you enjoy life?	Rarely-Occasionally	94	64.0
To what extent do you feel your life to be meaningful?	Not at all-Rarely	99	66.0

**Figure 7:**
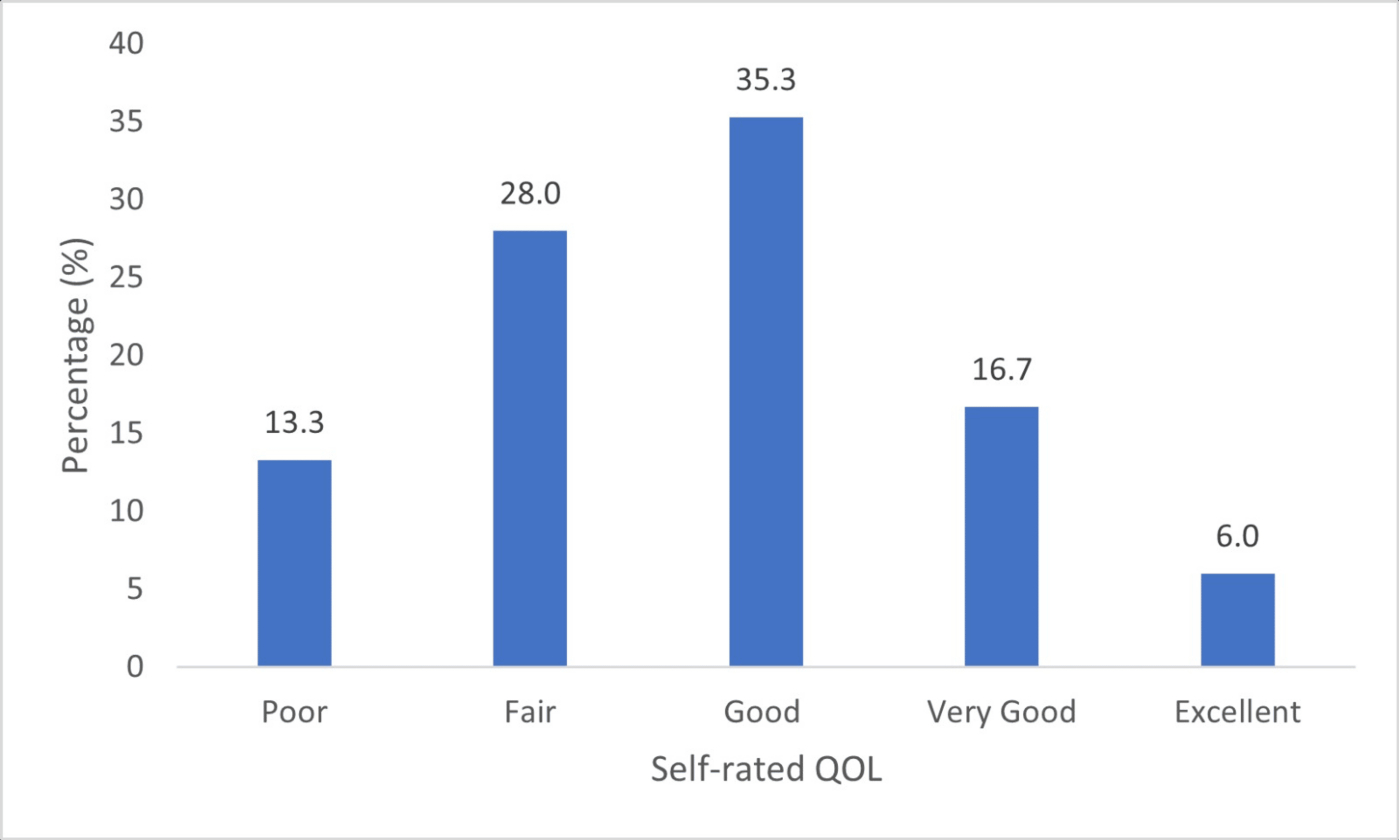
Self-rated QoL QoL: Quality of life

The leading response to each of the four items that were meant to assess caregiver QoL is presented in Figure 7.

Caregivers who lived with the patient had significantly higher mean QoL scores than those who did not (p = 0.040), as defined by the components used to measure it (Figure 7).

Correlations among stress, social well-being, economic well-being, and health status indicators

All bivariate correlations among stress, social well-being, economic well-being, and health status indicators were significant (Table 3). Specifically, QoL was negatively correlated with psychological, social, and economic factors.

**Table 3:** Correlations among stress, social well-being, economic well-being, and health status indicators **Statistically significant at the 5% level (p < 0.05) QoL: Quality of life

Items	Type of scores				
Type of scores	Stress	Social		Financial	QoL
Stress	1	0.729**		-0.289**	-0.004
Social	0.729**		1	-0.134	-0.011
Financial	-0.289**		-0.011	1	-0.137
QOL	-0.004		-0.137	-0.137	1

The data also showed that stress decreased with improved financial well-being.

## Discussion

The majority of caregivers were women (n = 82, 54.7%), aged between 36 and 75, with the highest representation in the 36-45 (n = 28, 18.7%), 46-55 (n = 44, 29.3%), and 56-65 (n = 31, 20.7%) age groups. Most caregivers had a tertiary-level education (n = 82, 54.7%) and were employed fulltime (n = 104, 69.3%). A similar demographic profile was observed in a study conducted by Edwards et al. [12], which discovered that most caregivers were women under 45 years old, employed, and possessed a college degree.

Regarding the relationship to the patient, 35.3% (n = 53) of caregivers were elderly children, 26.0% (n = 39) were spouses, and 13.3% (n = 20) were siblings. More than half (n = 83, 55.3%) of the caregivers lived with their patients, and 69.3% (n = 104) provided full-time care. Similarly, Kari et al. [13] discovered that 58% of caregivers were fathers, 25% were mothers, and 17% were other caregivers.

Most caregivers (n = 79, 52.7%) reported having at least one health condition. Additionally, 32 (21.5%) required medical checkups, and 36 (24.2%) reported increased alcohol consumption since becoming caregivers. These findings align with those of Ebadi et al. [5] and Llamas-Ramos et al. [14], who reported that caregiving affects physical health by impairing the quantity and quality of sleep, causing fatigue, increasing musculoskeletal disorders, and causing high levels of pain. Other studies have highlighted that female caregivers, in particular, report adverse physical and psychological health profiles, with a poorer QoL [15].

Additionally, more than 50% of the caregivers (n = 80, 53.3%) took the patient for dialysis three times per week, whereas 30 (20%) did so four times weekly. This responsibility can be demanding because travel time ranges from 45 minutes to three hours. In addition, regardless of their physical presence at the dialysis center, their caregiving responsibilities extend beyond the facility. Consequently, caregivers have reported additional problems, such as getting sick easily (n = 61, 40.6%) and burnout/exhaustion from stress (n = 101, 67.3%). Similarly, Schulz et al. [8] reported that caregivers admitted to missing personal medical appointments and developing unhealthy eating habits, particularly those from lower socio-economic backgrounds. Farzi et al. [16] also revealed that reducing the commuting rate lowered the number of caregiving hours and eased the caregiver burden.

Our study revealed major challenges to the caregivers’ social lives, with the most commonly reported being “hard to have a good work-life balance” (n = 106, 70.7%), followed by “unable to attend many social events taking care of the patient” (n = 93, 62.0%). Nkuranyabahizi et al. [17] reported similar findings, with most caregivers unable to balance their duties and activities of daily living, particularly those caring for patients with ESRD. Health Experiences Research Canada [18] also reported that caregivers felt exhausted or busy to be socially active, leading to a reduction in their friends and ultimately isolation. Other studies have reported that caregivers can be overwhelmed owing to caregiving demands (e.g., end-of-life care in ESRD) or a lack of resources [19]. Statistical analysis revealed significant pairwise differences in mean scores for four demographic variables: relationship to the patient (p = 0.005), caregiver age group (p = 0.025), main responsibility for taking patients to dialysis (p = 0.037), and caregiver’s highest level of education (p = 0.005).

The economic impact was also significant. Caregivers worry about financing (n = 102, 68.0%), cannot afford personal items (n = 83, 55.3%), and 66% (n = 99) cannot afford to send patients undergoing dialysis to a nursing home. Ninety-nine (66.0%) reported having to make changes to work hours and wished other family members would contribute financially (n = 107, 71.3%). These findings are consistent with that of the study by Abebe et al. [20], in which respondents identified the loss of financial capability and other assets, such as loss of job owing to absenteeism and incapacity to purchase essential household consumables, as economic burdens. Additionally, Ma et al. [21] attributed significant expenses primarily related to dialysis, laboratory tests, and hospitalization expenses, with drug costs accounting for 50% of the economic burden faced by patients undergoing dialysis and their families.

Burnout was prevalent (n = 101, 67.8%) and could be associated with exhaustion from the stress of having to take patients to dialysis sessions, with the majority being three times weekly (n = 83, 53.3%), as well as having full-time jobs (n = 103, 69.3%). Among psychological symptoms, feeling nervous, anxious, or on edge was most common (n = 115, 76.7%), followed by loss of personal time (n = 86, 57.3%). A significant proportion of respondents also reported feeling depressed (n = 49, 32.7%). Adejumo et al. [22] highlighted elevated anxiety (31.6% vs. 5.3%) and depression (31.6% vs. 3.5%) among caregivers compared with their counterparts. Caregivers also reported “I feel lonely as a result of taking care of the patient” (n = 24, 16.0%). Ekwall et al. [23] reported similar feelings of loneliness and social life. Our study revealed suicidal tendencies among 6.7% of caregivers. This value is within the broad range (2.7%-71%) reported by O’Dwyer et al. [24] who discovered that a significant proportion of caregivers experienced suicidal ideation. Self-reported suicidal ideation [25], which can be understated, reflects the trauma that caregivers undergo.

Global self-reported health status scores revealed that the majority of caregivers rated their QoL as “less than good” (n = 62, 41.3%). Moreover, when asked “To what extent do you feel life to be meaningful,” most responded either “Rarely” or “Not at all” (n = 99, 66.0%). This finding supports that of Sajadi et al. [4], who reported a poorer QoL among caregivers than in the general population.

Recommendations

Targeted interventions are needed to address caregiver issues in multiple domains without significantly affecting QoL. We recommend expanding supportive services for primary caregivers in public and private healthcare facilities. These programs should be tailored to address the psychosocial and economic burdens of caregivers and patients undergoing dialysis. These initiatives include implementing counseling services, support groups, and improved access to psychiatric care, alongside financial assistance such as subsidies for medical expenses, transportation support, and caregiver allowances. Furthermore, workplace policies should be adapted to provide greater flexibility. Accommodations such as adjustable schedules, telecommuting options, and unpaid leave should be encouraged to enable caregivers to effectively balance their caregiving responsibilities with their employment commitments. These measures are essential for supporting caregivers and enhancing their ability to provide quality care while maintaining their well-being.

Limitations 

The sample size was relatively small, especially for subgroup analyses. The participants relied on memory, which is subjective and inaccurate. In addition, inaccuracy may have resulted from a non-caregiver completing the questionnaire on behalf of the primary caregivers. As the study utilized an online platform, participants were limited to computer-literate caregivers.

## Conclusions

The results of this study showed significant socio-economic and psychological burdens faced by caregivers of patients on dialysis. More than half of caregivers (n = 80, 53.3%) took the patient for dialysis three times per week, which has led to more than 50% of caregivers having difficulty maintaining a work-life balance and attending social engagements. The majority also experienced economic hardships, worrying about finances (n = 102, 68.0%) and adjusting their work hours (n = 99, 66.0%). Depressive symptoms remained high (n = 49, 32.7%) but were significantly surpassed by feelings of anxiety (n = 115, 76.7%) and burnout (n = 102, 68.0%). More than half of the respondents reported no comorbidities; however, the majority claimed that they had been "getting sick easily" since becoming caregivers. These factors all impact the QoL. These findings underscore the need for targeted interventions to support caregivers and improve their overall QoL.

## Appendices

Questionnaire

Dear Willing Participant,

This study explores the Bio-psycho-socio-economic impact on the primary caregivers of Dialysis Patients. We are grateful for you completing the questionnaire as already consented to. We thank you for your participation in this study.

If you have any questions or concerns, please contact our supervisor.

●  Dr. Mandreker Bahall (Public health specialist)

●  Phone no: 763-6608

SECTION 1/8 - LOCATION OF DIALYSIS

Where does the patient receive Dialysis? (Please tick off one option)

North-West

□   Port-of-Spain General Hospital

□   West Shore Medical Private Hospital

□   Medcorp Ltd

North-Central

□   Mt Hope Hospital

□   St. Augustine Private Hospital

□   Reign Medical Clinic

□   Nova Medical Centre

□   Purivo Dialysis Centre

South

□   San Fernando General Hospital

□   RenoPure Medical Centre

□   Spectra Renal Care Dialysis Centre

□   Princes Town Dialysis Centre

East

□   Sangre Grande Hospital

□   Trinidad Dialysis

Centre Ltd (Sangre Grande)

SECTION 2/8 - PATIENT INFORMATION

For this section, please fill out the patient's information to the best of your knowledge.

1.      How old is the patient? □ Less than 18 years old □ 18 - 25 years old □ 26 - 35 years old □ 36 - 45 years old

□ 46 - 55 years old □ 56 - 65 years old □ 66 - 75 years old □ Older than 75 years

2.      What is the patient's sex? □Male □ Female

3.      What is the patient's ethnicity? □ Afro-Trinidadian □ Indo-Trinidadian □ Caucasian (White) □ Asian □

Mixed □ Other

4.      Where in Trinidad, does the patient live? □ North □ South □ East □ West □ Central

5.      What is the highest level of education the patient has attained? □ University Degree □ Secondary School

□ Primary School □ They learnt a trade/skill □ I am unsure

6.      Is the patient currently working at a job? □ Yes. They are working Full Time □ Yes. They are working Part-Time □ No. They are Unemployed right now □ No. They are Retired □ I am unsure

7.      What is the average amount of money the patient gains each month? □ About TT$2,500 or less

□ TT $2,501 - 5,000 □ TT $5,001 - 7,500 □ TT$7501 - 10,000 □ Over TT $10,000 □ I am unsure

8.      Where does the patient gain their monthly income? □ They gain a salary from working □ I or a family member finances them □ They use their personal savings □ They receive pension/NIS □ They are supported by Social Welfare or a Disability Income □ I am unsure

9.      What is the patient's marital status? They are

□ Single □ Married □ Living together with a spouse (Common Law) □ Divorced □ Widowed

□ Living Separate from their husband/wife □ I am unsure

10. What is the patient's religion? □ Christian □ Muslim □ Hindu □ They do not follow a religion

□ I am unsure □Other

11. Does the patient have any medical conditions, apart from needing dialysis? (Please select as many options that apply)

□ No. They have no other medical conditions □ They have Diabetes (High blood sugar) □ They have Hypertension (High blood pressure) □ They have high Cholesterol □ They have Heart problems □ They have cancer □ They have a disability □ I am Unsure □ Other

12. What type of Dialysis is the patient receiving? □ Haemodialysis - blood enters a machine to be cleaned

□Peritoneal Dialysis - a drips/IV bag is attached to the patient's belly □ I am unsure

13. How long does a dialysis session take for the patient? □1 - 2 hours □2 - 3 hours □3 - 4 hours □Over 4 hours

14. How many days a week does the patient receive dialysis? □ Once per week □ 2 days per week □ 3 days per week □ 4 days per week □ 5 days per week □ 6 days per week □ Every day per week

15. How long has the patient been on dialysis thus far? □ Less than 1 year □ 1 - 3 years □ 3 - 5 years

□ 5 - 7 years □ 7 - 9 years □ Over 9 years

SECTION 3/8 - CAREGIVER INFORMATION

This section and all the sections onward are focused on YOU as the primary caregiver of the dialysis patient. Please answer each question to the best of your ability.

1.      Are you the MAIN caregiver responsible for taking care of the dialysis patient? □ Yes □ No

2.      What is your relationship to the patient? I am the                    of the patient.

□ Husband/Wife □ Parent □ Son/Daughter □ Brother/Sister □ Grandson/Granddaughter

□ Friend □ Paid/Hired Caregiver □ Other:

3.      What is your age? □ Less than 18 years old □ 18 - 25 years old □ 26 - 35 years old □ 36 - 45 years old

□ 46 - 55 years old □ 55 - 65 years old □ 65 - 75 years old □ Older than 75 years old

4.      What is your sex? □ Male □ Female

5.      What is your Ethnicity? □ Afro-Trinidadian □ Indo-Trinidadian □ Caucasian (White) □ Asian

□ Mixed □ Other:

6.      Do you live with the dialysis patient? □ Yes □ No

7.      What is your highest level of education? □University Degree □Secondary School □Primary School □I learnt a trade/skill

8.      Are you currently working? □ Yes. I am working full-time □ Yes. I am working part-time

□ No. I am currently unemployed □ No. I am retired

9.      What is your average monthly income? □ TT$2,500 or Less □ TT $2,501 - 5,000 □ TT $5,001 - 7,500

□ TT$7501 - 10,000 □ Over TT $10,000

10.  What is your marital status? □ Single □ Married □ Living with someone (common law) □ Divorced

□ Widowed □ Living Separate from my husband/wife

11.  What is your religion? □ Christian □ Muslim □ Hindu □ I do not follow a religion □ Other:

12.  Are you the MAIN person responsible for carrying the patient to the dialysis centre? □Yes.

□ No. Someone else is responsible for carrying them □ No. The patient goes for dialysis for themself

13.  How far is the dialysis center from your home? □ It is very close by; it takes less than 45 minutes to reach □

About 1 hour □ 2 hours □ 3 hours □ More than 3 hours

14.  Do you wait/stay with the patient while they are receiving dialysis? □Yes □ No. I drop them off, then return to pick them up □ Sometimes I stay, but sometimes I leave

*SECTION 4/8 - MEDICAL HISTORY OF CAREGIVE*R

In this section, Please Select Yes or No for each of the following medical conditions:

1.      Do you have Diabetes (High blood sugar)? □ Yes □ No

2.      Do you have Hypertension (High blood pressure)? □ Yes □ No

3.      Do you have high cholesterol? □ Yes □ No

4.      Do you have Heart Disease/Blocked arteries? □ Yes □ No

5.      Have you gained more than 8 pounds in the last 3 months? □ Yes □ No

6.      Have you lost more than 8 pounds in the last 3 months? □ Yes □ No

7.      Do you get sick easily? □ Yes □ No

8.      Are you experiencing increased alcohol consumption? □ Yes □ No. My drinking has not increased

□No. I do not drink alcohol at all

9.      Do you smoke? □ Yes □ No

10.  Are experiencing any self-harm or suicidal thoughts? □ Yes □ No

11.  Are you experiencing burnout/exhaustion from stress? □ Yes □ No

12.  Have you had to see a doctor to check your health, since you started caregiving for the patient?

□ Yes □ No

SECTION 5/8 - PSYCHOLOGICAL FACTORS

For this section, please select the option that best describes how you feel during your time as a caregiver.

1.      I feel nervous, anxious, or on edge

Not at all    □ 0  □ 1  □ 2  □ 3     Everyday

2.      I worry about too many different things at once

Not at all    □ 0  □ 1  □ 2  □ 3     Everyday

3.      I have trouble relaxing

Not at all    □ 0  □ 1  □ 2  □ 3     Everyday

4.      I feel like I get easily annoyed recently

Not at all    □ 0  □ 1  □ 2  □ 3     Everyday

5.      I feel like something bad might happen all the time

Not at all    □ 0  □ 1  □ 2  □ 3     Everyday

6.      I feel down, depressed, or hopeless

Not at all    □ 0  □ 1  □ 2  □ 3     Everyday

7.      I have little interest or pleasure in doing things

Not at all    □ 0  □ 1  □ 2  □ 3     Everyday

8.      I have trouble falling asleep, or I am oversleeping

Not at all    □ 0  □ 1  □ 2  □ 3     Everyday

9.      I have trouble concentrating

Not at all    □ 0  □ 1  □ 2  □ 3     Everyday

10.  I have noticed that I am moving and/or speaking less or slower

Not at all    □ 0  □ 1  □ 2  □ 3     Everyday

11.  Do you feel strained/stressed by taking care of the patient?

Not at all    □ 0  □ 1  □ 2  □ 3     Everyday

12.  Do you feel angry when you are around the patient?

Not at all    □ 0  □ 1  □ 2  □ 3     Everyday

13.  Do you feel like you have less personal time and space, since you started taking care of the patient?

Not at all    □ 0  □ 1  □ 2  □ 3     Everyday

14.  Do you feel like you cannot leave the patient alone for too long?

Not at all    □ 0  □ 1  □ 2  □ 3     Everyday

15.  Do you feel like you could be doing more/better in caring for the patient?

Not at all    □ 0  □ 1  □ 2  □ 3     Everyday

SECTION 6/8 - SOCIAL FACTORS

For this section, please select the option that best describes how you feel during your time as a caregiver.

1.      I have good social support from family and/or friends □ Yes □ No

2.      I feel lonely as a result of taking care of the patient. □ Yes □ No

3.      I am not able to attend many social events and activities, because I am busy taking care of the patient.

□ Yes □ No

4.      Taking care of the patient has changed my relationship with my closest relationships. □ Yes □ No

5.      I have found it hard to have a good work-life balance since I started caregiving. □ Yes □ No

6.      I have had spent less time on my hobbies (things I do for fun) due to my responsibility of caregiving.

□ Yes □ No

7.      I have experienced judgement from others due to my role as a caregiver. □ Yes □ No

SECTION 7/8 - ECONOMIC FACTORS

For this section, please select the option that best describes how you feel during your time as a caregiver.

1.      I worry about my finances, as I have had to help pay for the care of the patient at times.

□ Yes □ No

2.      I wish other members of the family would help contribute financially to taking care of the patient.

□ Yes □ No

3.      I feel that I cannot sufficiently afford to pay for food, clothes, or vacations for myself or my family, due to caregiving for the patient. □ Yes □ No

4.      I feel that my family argues more about money now than we did before I was responsible for caregiving.

□ Yes □ No

5.      I feel that I cannot send the patient to a nursing home because it is too expensive.

□ Yes □ No

6.      I have had to make changes to my work hours or job, to be able to care for the patient.

□ Yes □ No

SECTION 8/8 - QUALITY OF LIFE

For this section, please select the option that best describes how you feel during your time as a caregiver.

1.      How would you rate your quality of life?

Very Poor            □ 1  □ 2    □ 3    □ 4    □ 5     Very Good

2.      Are you satisfied with your health?

Very Dissatisfied    □ 1    □ 2    □ 3    □ 4    □ 5    Very Satisfied

3.      Do you feel any physical pain in your daily activities?

Not at all    □ 1     □ 2    □ 3    □ 4    □ 5    An Extreme Amount

4.      Do you have enough energy for everyday life?

Not at all    □ 1     □ 2    □ 3    □ 4    □ 5    Completely

5.      How safe do you feel in your environment?

Not at all    □ 1     □ 2    □ 3    □ 4    □ 5    Extremely Safe

6.      How satisfied are you with your personal relationships?

Very Dissatisfied    □ 1    □ 2    □ 3    □ 4    □ 5    Very Satisfied

7.      How satisfied are you with your sex life?

Very Dissatisfied    □ 1    □ 2    □ 3    □ 4    □ 5    Very Satisfied

8.      Have you enough money to meet your needs?

Not at all    □ 1     □ 2    □ 3    □ 4    □ 5    Completely

9.      How much do you enjoy life?

Not at all    □ 1     □ 2    □ 3    □ 4    □ 5    An Extreme Amount

10.  To what extent do you feel your life to be meaningful?

Not at all    □ 1     □ 2    □ 3    □ 4    □ 5    An Extreme Amount

                                          End of Questionnaire
